# Both Wrong and Right Methods Explained

**Published:** 1921-02-26

**Authors:** 


### THE MEDICAL USES OF OXYGEN.

### Both Wrong and Right Methods Explained.

At a time when empiricism in the use of drugs is discouraged, and none are accepted unless they can show some definite and actual effect, it is rather a matter for wonder that a drug of such proved value as oxygen should meet with such meagre recognition.

There is doubtless some reason for this, and it is probable that the cumbersome nature of the cylinders, and their malicious habit of being empty when most urgently required is one of the most important. There is also the dislike which we all develop to methods of treatment which, being used most often in almost hopeless cases, become associated in our minds with a fatal result. If this is the case it is a pity, for by neglecting to use oxygen the physician sacrifices a very valuable therapeutic agent, by the intelligent use of which a great deal of good can be accomplished.

To stand a cylinder of oxygen beside the patient's bed and, pinning the end of a rubber tube to the bedclothes somewhere near the patient's mouth, to blow a casual stream of gas at him, is not in any way an intelligent method, nor is this improved by fixing a glass funnel to the end of the tube.

### Necessary Precautions.

There are certain definite precautions which must be taken before the gas from the cylinder can be delivered to the patient. As it leaves the cylinder the gas is passing from a very high pressure to the ordinary atmospheric pressure, and in expanding it loses great quantities of heat, becoming very cold and quite unsuitable for respiration. It is also stored pure and dry, and as it expands, what little moisture there is, is even more diluted; this intense dryness renders the gas unsuitable for respiration. To overcome these drawbacks the gas on leaving the cylinder must be passed through a bottle of hot water, and, so that both warmth and moisture may be imparted in one process, and also the rate of flow gauged, it is convenient to let the gas bubble up through the water. The addition of alcohol in the form of brandy or whisky to this hot water is often advocated, and in patients who need stimulating this is often a very valuable addition. From this wash bottle, which should be stood in a bowl of water kept at a temperature of between 80° to 100° Farenheit, the oxygen is led to a mask which can be fixed to the patient's face, or to a nasal catheter. During the war many types of mask were designed, as oxygen formed one of the staple methods of treatment for soldiers who had been gassed. Most of these types can claim considerable efficiency, but probably an ordinary catheter passed *via* the nose is as good. It is very important that the mask or catheters should be fixed firmly, for cyanosed patients, and all who are having to struggle for their breath, are not infrequently restless, and if the delivery tube is fixed to the bedclothes or the pillow it frequently happens that the gas is being directed only on to the back of the patient's head.

### The True Value of Oxygen.

Also the true value of oxygen should be clearly understood if it is to be used with success. There is a prevalent belief that oxygen is a stimulant, in the way that caffeine and strychnine are, and that after half-a-dozen deep breaths of oxygen the normal man will become sufficiently encouraged to rebuke his cook, or embark on any other similarly hazardous enterprise. This, it is true, is only the impression among the lay public, but it has materialised through the profession so must exist there too. Oxygen has not this effect to any appreciable effect. Its chief value is that it rests the heart and the lungs. The body for its existence requires a definite minimum amount of oxygen, and this has to be pumped to it by the heart. The blood to get this oxygen requires that a certain volume of air must pass through the lungs, and this air contains 20 per cent. of oxygen. If both heart and lungs are being over-driven to keep the minimum of oxygen in circulation, there is relief when the quantity of air breathed contains an increased percentage of oxygen.

### Suitable Cases.

As to the types of cases most benefited by the administration of oxygen, they fall into three classes:—

Class I contains those cases in which the power of the lung to absorb oxygen is reduced either by complete occlusion of part of the lung by fluid in it, pressure by fluid or air in the pleura, or inflammatory processes of the mucous membrane lining the lungs rendering them impervious to gases.

Class II consists of cases in which the heart is unable without excessive strain to keep up a supply of blood sufficient to satisfy the demands of the tissues for oxygen.

Class III contains cerebral and uræmic cases in which the action of either the heart or the lungs is disorganised by asphyxia or by pressure on their centres in the brain.

In administering oxygen it is usual to give the gas for say five minutes at a time, and half hourly; but with the use of a suitable mask and a wash bottle through which the quantity of gas can be gauged it is possible to keep the patient continuously supplied with just the right quantity of oxygen to keep him comfortable, and a good colour, and this without using nearly as much oxygen as is allowed to run to waste by more haphazard methods. This continuous supply of oxygen will often be found to *rest* the patient to a really remarkable extent, and keep him a satisfactory pink instead of a dirty bluish purple.

